# The homeless, seizures, and epilepsy: a review

**DOI:** 10.1007/s00702-023-02685-8

**Published:** 2023-08-22

**Authors:** Rita Pontes Silva, João Gama Marques

**Affiliations:** 1 Clínica Universitária de Psiquiatria e Psicologia Médica, Faculdade de Medicina, Universidade de Lisboa, Lisbon, Portugal; 2 Consulta de Esquizofrenia Resistente, Hospital Júlio de Matos, Centro Hospitalar Psiquiátrico de Lisboa, Lisbon, Portugal

**Keywords:** Homeless, Seizure, Epilepsy

## Abstract

This review aims to estimate the prevalence of seizures and epilepsy among homeless people in current literature as well as understand the main adversities that this group withstands. We conducted a search for “epilep*”, “seizur*”, and “homeles*” in titles and abstracts of articles in PubMed. Overall, 25 articles met the final inclusion criteria and warranted analyses. This study suggests that the prevalence of epilepsy in the homeless population is between 2 and 30%, whereas the prevalence of homelessness in people with epilepsy is between 2 and 4%. Every study included in this review corroborates the increased prevalence of seizures and epilepsy among the homeless, which puts them at risk for worse outcomes related to this condition and numerous associated comorbidities. Further evidence is needed to clarify the distinction of primary and secondary seizures in this group, which shows a high rate of confounding factors for seizures like substance abuse or withdrawal and head injury, and to decrease the burden of epilepsy and homelessness in an already resource-deficient community.

## Introduction

### Epilepsy

Epilepsy is a chronic neurological disease characterized by recurrent episodes of involuntary movement called seizures, that may be focal (restrained to only a specific part of the body) or generalized (affecting the entire body), and might have concurrent loss of consciousness and/or bowel or bladder control. It is included by the World Health Organization (WHO) in the International Classification of Diseases—11th Revision (ICD-11) with codes 8A60 to 8A6Z (WHO 2022).

Seizures are the physical representation of excessive and disorganized electric impulses of the neurons, resulting in a wide range of clinical manifestations: from minute-long lapses of attention (absence seizures) to prolonged life-threatening convulsions (tonic–clonic seizures). One seizure does not make a diagnosis of epilepsy. Up to 10% of people worldwide have one isolated seizure in their lifetime (WHO 2022).

**Fig. 1:**
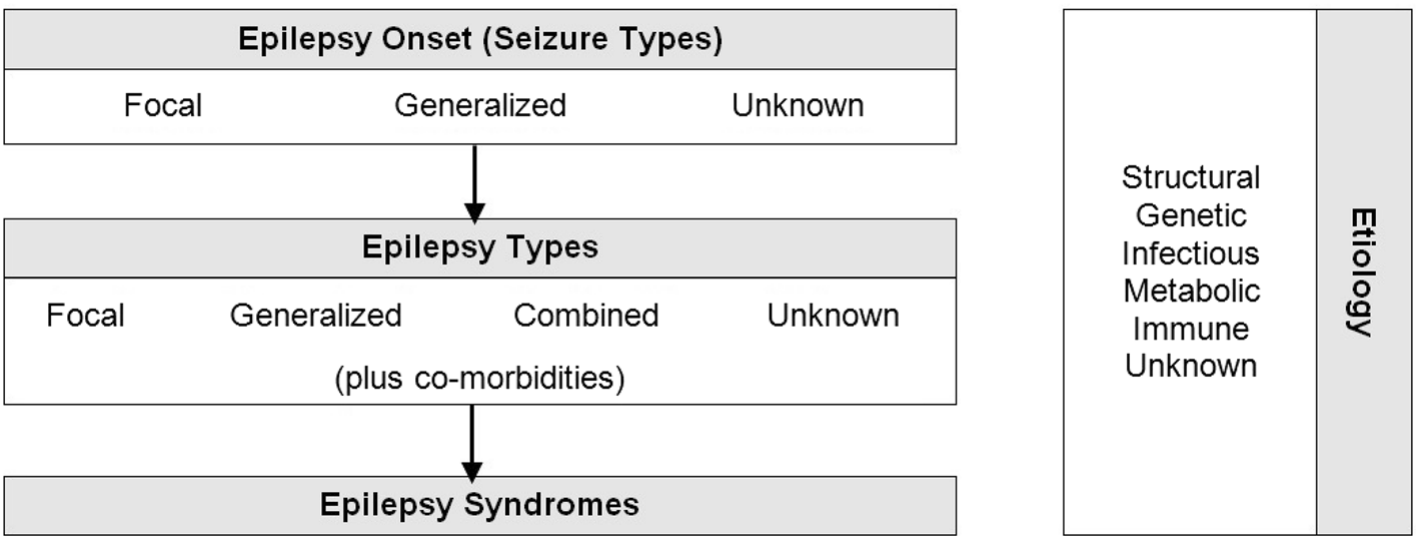
Classification of Epilepsy (adapted from ILAE)

The International League Against Epilepsy (ILAE) defined epilepsy as the presence of one of the following: two or more unprovoked seizures more than 24 h apart, one unprovoked seizure and a high individual risk of seizure recurrence (e.g., after head injury, stroke, central nervous system infections) or a diagnosis of an epilepsy syndrome (Scheffer et al. 2017). The diagnosis relies primarily in clinical history taking, physical examination and electroencephalography (EEG). This disorder may be further characterized as to the success rate of treatment as pharmacoresponsive or not, as appropriate. The most recent classification of epilepsy by the ILAE (Scheffer et al. 2017) is represented in Fig. 1.

According to the WHO (2022), epilepsy is one of the most common neurological diseases worldwide with 50 million estimated people and 5 million new diagnoses each year. Near 80% of people with epilepsy (PWE) live in low and middle-income countries, with 139 per 10,000 people diagnosed each year, opposed to 49 per 100,000 in high-income countries. This fact may be precipitated by the increased risk of conditions such as infections (e.g., neurocysticercosis), road traffic injuries, birth-related complications, and absence of accessible care, medical infrastructures or public health prevention programs endemic to these countries. There are also numerous social barriers to ideal care and overall health outcomes for people with epilepsy, defined as upstream factors of illness or Social Determinants of Health (SDoH) (Szaflarski 2014).

The WHO defines SDoH as the conditions in which people are born, grow, live, work and age (…) shaped by the distribution of money, power and resources at global, national and local levels. Such SDoH, who have been widely proved to interact and interfere with one’s health and overall quality of life, have not been extensively described in epilepsy or PWE (Sirven et al. 2022). In 2014, Szaflarski stated that the core SDoH in this population where socioeconomic status, race/ethnicity, age, gender, contextual factors (social stigma and discrimination), living and employment situation, treatment adherence, social support and factors intrinsic to each health system. All were associated with poorer epilepsy care and outcomes. One group that embodies the worst outcomes in most of the listed SDoH is the homeless population.

### Homelessness

Homelessness is considered the most severe form of social impairment with a great burden of social stigma (Gabriel et al. 2020). At the WHO’s ICD-11 homelessness received the code QD71.0. The European Typology of Homelessness and Housing Exclusion (ETHOS) classified the different types of housing situations, as illustrated in Table 1, adapted from the *Fédération Européenne d'Associations Nationales Travaillant avec les Sans-Abri* (*FEANTSA* 2017), also known as the European Federation of National Organisations working with the Homeless. Homeless people describe substantially worse overall health than housed people (Lewer et al. 2019). This group has a three-fold risk of reporting various conditions, such as, among others, epilepsy and seizures (Gelberg et al. 1990; Lewer et al. 2019). It is not uncommon for people to become homeless because of their epilepsy (Laporte et al. 2006) or to have a new diagnosis of epilepsy while living homeless, secondary to substance abuse or Traumatic Brain Injury (TBI) (Talan et al. 1998; Svoboda and Ramsay 2014; Lewer et al. 2019). Seizures are the second most frequent neurological cause Emergency Department (ED) admission in this group (Rosendale et al. 2019).

**Table 1:** Classification of homelessness (adapted from *FEANTSA*)

Conceptual category	Operational category	Living situation
Roofless	People living rough	Public or external space
	People in emergency accommodation	Overnight shelters
Houseless	People in accommodation for the homeless	Homeless hostels Women shelters Temporary accommodation Transitional accommodation Refugee accommodation
	People living in institutions	Health care institutions Penal institutions
Inadequate housing	People living in non-conventional dwellings	Mobile homes Non-conventional buildings Temporary structures

### Epilepsy and homelessness

Doran et al. (2021a, b) suggest that seizures are often inadequately managed because of comorbid substance abuse and mental illness, treatment non-adherence and unstructured specialist care (all highly prevalent in homeless populations), resulting in increased morbidity and mortality. The combination of homelessness and epilepsy generates one large stigmatized and neglected niche of people who are underrepresented in current literature and are being mismanaged in clinical settings. The aim of this review was to study the relation between epilepsy and homelessness.

## Methods

**Fig. 2:**
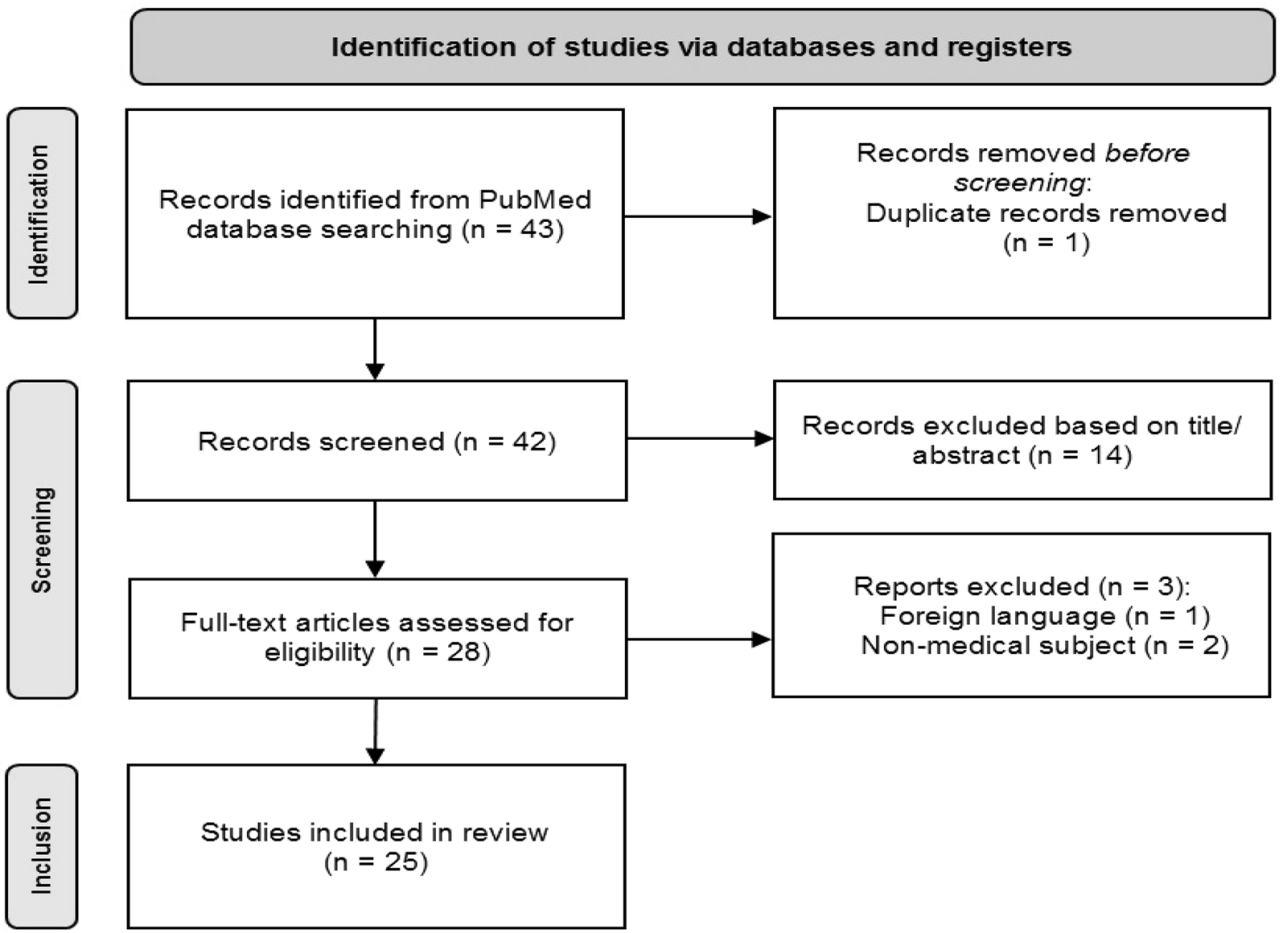
Flowchart for article selection in the review regarding the homeless, seizures and epilepsy

A search was conducted for all-research types regarding the keywords “homeless*” plus “seizur*” or “epilep*” to titles and abstracts of articles. All articles that resulted from this search were selected. Relevant articles underwent data extraction and analysis. Articles were excluded if they were not written in English, if they were off topic, duplicates or not accessible. Using the PubMed online database, on April 9th 2022, we found 43 studies, that were included in this review. Apart from the database mentioned, relevant organizations websites were consulted, from pertinent organizations, such as the ICD, the WHO, the ILAE and the *FEANTSA*. The primary outcome was defined as the prevalence of epilepsy and seizures in the homeless population. Three secondary outcomes were set: epilepsy as a risk factor for homelessness, homelessness as a risk factor for epilepsy and disease burden. The full electronic search history is shown in Fig. 2.

## Results

Relevant articles underwent data extraction and analysis where conclusions of this review are drawn from. There were 43 results identified and 1 duplicate was excluded before screening. Then, 42 records were screened and 14 were excluded based on title and abstract review. The remaining 28 full-text articles were reviewed and 3 were excluded based on exclusion criteria: off-topic articles (*n* = 2) or non-English studies (*n* = 1). Overall, 25 articles met final inclusion criteria and warranted analysis: retrospective cohort studies (*n* = 11), cross-sectional studies (*n* = 11) and reviews (*n* = 2). Table 2 presents the general characteristics of included studies.

**Table 2:** Outcomes assessed, for every article included in this review

Author (year)	Study design	Country	Sample (*N*)	Admission	Mortality
Ayano et al. (2017)	Cross-sectional	Ethiopia	456		
Bowe and Rosenheck (2015)	Cross-sectional	USA	638,451	X	
Doran et al. (2021a, b)	Cohort	Ireland	46	X	
Doran et al. (2021a, b)	Cohort	Ireland	88	X	
Gabriel et al. (2020)	Cohort	Portugal	333		
Gelberg et al. (1990)	Cross-sectional	USA	464	X	
Hanzlick and Lazarchick (1989)	Cross-sectional	USA	18		X
Hwang et al. (2008)	Cross-sectional	Canada	904		
Hwang (2001)	Review	Canada	8000	X	X
Laporte et al. (2006)	Cross-sectional	France	592		
Lennard et al. (2022)	Cohort	England	450	X	X
Lewer et al. (2019)	Cross-sectional	England	14,696		
Moss et al. (2021)	Cohort	England	90,941	X	X
O'Farrell et al. (2016)	Cross-sectional	Ireland	2051	X	
O’Toole et al*.* (2007)	Cohort	USA	326	X	
Ramamurthy et al. (2021)	Cohort	USA	122	X	
Rimmer et al. (2007)	Cross-sectional	USA	32	X	
Rosendale et al. (2019)	Cross-sectional	USA	1,082,347	X	X
Sirven et al. (2022)	Cohort	USA	5965		
Steel et al*.* (2021)	Cohort	USA	209,151	X	
Subedi et al. (2022)	Cohort	USA	2658	X	
Svoboda and Ramsay (2014)	Cohort	Canada	169	X	
Talan et al. (1998)	Cross-sectional	USA	900,000	X	
Wood (1989)	Cross-sectional	USA	200		

USA: United States of America

### Prevalence of epilepsy in homeless people

Eight studies reported a prevalence of seizures/epilepsy in homeless people between 1.75 and 30% (Gelberg et al. 1990; Laporte et al. 2006; Svoboda and Ramsay 2014; Ayano et al. 2017; Lewer et al. 2019; Rosendale et al. 2019; Doran et al. 2021a, b; Subedi et al. 2022).

Laporte et al. (2006) found a correlation between the length of homelessness situation and seizures prevalence: those living with no fixed abode for less than 2 years reported 9.6% of seizures; people who were homeless for more than two years had a higher prevalence of 20.4%.

Svoboda and Ramsay (2014) estimated that people entitled chronically homeless with alcoholism represented 62% of seizure episodes while the rest of the general homeless population reported only 11.5%.

Ayano et al. (2017) studied a group of homeless people with observable psychopathology as well as concomitant seizure disorders, with a prevalence of 1.75%.

Doran et al. (2021a, b) reported prevalence of 30% (Doran et al. 2021a, b) regarded a small sample of 46 people, possibly not representative of the general homeless population.

Four other studies reported a prevalence of homelessness among PWE between 1.8 and 4% (Rimmer et al. 2007; Gabriel et al. 2020; Ramamurthy et al. 2021; Lennard et al. 2022).

### Epilepsy as a risk factor for homelessness

Gabriel et al. (2020) have recently published a study to assess social impairment in specific epileptic syndromes such as genetic generalized epilepsy (GGE) and mesial temporal lobe epilepsy with hippocampal sclerosis (MTLE-HS), a more severe and difficult-to-treat syndrome. They concluded that people diagnosed with benign epilepsy or, as ILAE’s preferred term, self-limited and pharmacoresponsive epilepsy, such as GGE, are less protected from homelessness (with 34.1% of people with no fixed abode) than people with MTLE-HS (with 0% homeless people recorded). The second group probably has been better aided by their families and equally been better managed by social workers, given the severity of their condition. The MTLE-HS group also reported experiencing more stigma than GGE people. (Gabriel et al. 2020).

Hwang et al. (2008) reported that, in a 70% of a given homeless population in Canada, the first TBI occurred before their first experience of homelessness, thus suggesting a probable causal connection between the two events.

### Homelessness as a risk factor for epilepsy

Talan et al. (1998) created and implemented a network for signaling and studying emerging infectious diseases in ED, where they are more likely to present, among the homeless people. One of the illnesses under investigation was neurocysticercosis, which is the most common parasitic infection of the brain in the United States of America (USA), with seizures as its most usual presentation.

Laporte et al. (2006), in a study with 592 homeless people, reported 40.7% of alcohol related seizures, suggesting that alcohol abuse is an important trigger for seizures and epilepsy in the homeless population. They also disclosed that, in almost half of the homeless people included in the study, the frequency of seizures increased after the onset of homelessness, mostly because of alcohol abuse.

Hwang et al. (2008) found a prevalence rate of mild to severe self-reported TBI of 53%, which represents almost five times the rate in general population. A younger age of onset and longer period of living in a homeless situation were identified as risk factors for TBI, as well as a personal history of seizures. Hwang et al. (2008) also proposed that most moderate to severe TBI were associated with significantly increased odds of seizures (odds ratio 3.2), psychiatric disorder or drug abuse.

Svoboda and Ramsay (2014) studied the incidence of TBI presentation to the emergency department (ED) in three distinct groups of people: low-income housed, homeless and alcoholic homeless. The rate of TBI was 17 times higher in the alcoholic homeless group (28%) than low-income housed (5%) and the homeless (3%), and groups combined, attesting to the dangers of alcohol abuse in those with housing instability.

Bowe and Rosenheck (2015), in a study of war veterans suffering from post-traumatic stress disorder (PTSD) and substance abuse disorder, discovered that these people were prone to homelessness as well as seizure disorders.

### Burden of seizures, epilepsy and homelessness

Hanzlick and Lazarchick (1989) stated that two thirds of a group of homeless people who died had used public health facilities prior to their deaths, most of which were directly or indirectly caused by alcohol misuse, an important cause of seizures, either when abused or in withdrawal.

O’Toole et al*.* (2007) postulated that homeless individuals diagnosed with chronic ambulatory care sensitive condition (ACSC), such as epilepsy, that were currently registered and monitored in ambulatory care were less likely to resort to the ED.

O’Farrell et al*.* (2016) reported a rate of 32.9% of seizure-related hospital admissions in the homeless population, while Rosendale et al. (2019) reported 19%.

Rosendale et al. 2019 reported other non-ACSC in the ED in homeless people like TBI and encephalopathy, both important cause and consequence of seizures.

Moss et al. (2021), in a comparative study with more than 16,000 homeless people, predicted that this sample group reported 1.79 times higher rates of overall annual hospital admissions, 2.08 times for ED admissions and 1.65 times for ACSC than housed participants. Among the ACSC more frequent among homeless people were seizures. Acute hospital admissions for seizures were 2.4 times more frequent in this group than in the housed comparison parcel (12% and 5%, respectively).

Doran et al. (2021a, b) conducted a study with 88 homeless individuals with seizures and a matching set of housed ones over a period of 5 years and concluded that, since the former group has a higher risk of having unwitnessed seizures with no reliable history of the event (and, as such, no reports of possible head trauma or duration of seizures), they are more likely to be subject to more invasive investigation of etiology and complications of the episode. Altogether homeless people underwent 250 computerized tomography (CT) brain scans in the 5-year time period of this study, with a mean of 3 scans per patient. Of all the CT scans performed with valid clinical indication (stroke, TBI, new seizure type and prolonged loss of consciousness) only 8% had a positive result for intracranial anomaly and were relevant to treatment, most frequently related to TBI. Nine people in the homeless group had 10 CT brain scans in the course of 5 years, six of which already had a formal diagnosis of epilepsy and most likely did not require a scan in the first place. Ten scans equal an exposure of radiation equivalent to 700 X-rays or 14 milliSievert (mSv). The maximal radiation dose for one patient in this time period was 44.8 mSv, which, while still being inferior to the supposed carcinogenic dose of 100 mSv, may still increase throughout the rest of a patient’s history of ED admissions in a lifetime, and reach a cumulative dose that puts them at risk for radiation-induced cancer.

Three different studies found that homeless people had an early readmission rate more than 2 times higher than the housed population (Rosendale et al. 2019; Lennard et al. 2022; Subedi et al. 2022). Rosendale et al (2019) and Subedi et al (2022) described epilepsy and homelessness are both risk factors for early hospital readmission. Rosendale et al. (2019) stated a two-fold risk for the same outcome. Subedi et al. (2022) reported 35% higher readmission rate for epilepsy in homeless people. Lennard et al. (2022) uncovered 12.78% readmissions for seizures, with minimum attendance of 2 and maximum of 12 readmissions over a four-year period, with nearly half of the admittees lacking a former prescription of antiseizure medication.

Sirven et al. (2022) studied the role of SDoH in epilepsy treatment delays and concluded that both homelessness and outpatient prescriptions increased the likeliness of a patient going untreated. Circa 40% of people went untreated and only 18% of PWE received timely treatment. Treatment delays in epilepsy increase the risk for seizure-related injuries (TBI, accidents and suicidality) and are responsible for a higher number of hospital admissions among the homeless people.

Hwang (2001) established an increased risk of mortality in younger than 25 years old homeless people up to 9 times for men and 31 for young women. The most frequent causes of death among this population are accidents, overdose, suicide and alcoholism related diseases.

Laporte et al. (2006) found a lower rate of epilepsy in homeless men older than 60 years, which is inconsistent with normal prevalence of epilepsy in this age group, and suggests an earlier, increased mortality for homeless people with epilepsy.

Rimmer et al. (2007) described that causes of epilepsy-related death are mostly preventable, such as unintentional injuries (falls, drowning, burns), suicide, prolonged seizures (*status epilepticus*) and Sudden Unexpected Death in Epilepsy (SUDEP).

O’Farrell et al. (2016) found an average age of death of 48.2 years in hospitalized homeless people.

Lewer et al. (2019) described mortality rates up to six times higher (Lewer et al. 2019) homeless people usually exhibit a life expectancy two decades shorter than the housed population.

Moss et al. (2021) compared the average life expectancy in England, 76 and 81 years for men and women, with a much shorter existence, among the homeless people, 45 and 43 years, respectively.

Lennard et al. (2022) reports a mortality rate of 19% in a retrospective study of 450 PWE.

## Discussion

This review suggests that the prevalence of epilepsy in the homeless population is between 1.75 and 30% whereas the prevalence of homelessness in PWE is between 1.8 and 4%. There is a consensus between every study included in this review that there is as increased prevalence of seizures and epilepsy among the homeless that puts them at risk for worse outcomes related to this illness and numerous associated comorbidities.

PWE have suffered with social stigma since medieval times, with demonic possession allegations associated with clonic seizure activity, until more recently to the eugenics movement, of the last two centuries. PWE have less educational opportunities and experience more difficulties in obtaining a driver’s license, pursuing some professions and acquiring life insurance, for example (WHO 2022). In some countries there are even laws that prohibit PWE to access restaurants, theaters and other public buildings, reflecting the stigma that these people still endure in the twenty-first century (WHO 2022). Social impairment in epilepsy deteriorates the overall quality of life of PWE, with negative repercussions in academic achievement, employment, monthly income, housing and personal relationships, disrupting their independence and negatively affecting all SDoH. PWE undergo stigma and social exclusion, leading to a life-threatening negative impact on all SDoH like loss of income, family integration and, ultimately, appropriate housing (Gabriel et al. 2020). Similarly, people living in homeless situation (the paradigm of extreme social impairment) are more predisposed to hostile environment and unfortunate living arrangements, such as head trauma and substance abuse, which are known causes of seizures and epilepsy (Hwang et al. 2008). Seizures limit one’s chances of overcoming homelessness in the same way as having no fixed abode worsens the disease burden of epilepsy (Laporte et al. 2006), suggesting a bidirectional causality between the two.

Apart from psychosocial impairment, PWE are also associated with psychiatric comorbidities predating seizure onset suggesting a bidirectional relationship and perhaps a common underlying mechanism for some psychiatric disorders and epilepsy (Hesdorffer et al. 2012). PWE have a two to three times higher incidence of psychotic disorders than the general population (Rai et al. 2012), especially schizophrenia-like psychosis mimicked by epilepsy itself (ictal, interictal and postictal psychosis) or as a side effect of antiseizure medications (Braatz et al. 2021). Secondary side effects of antiseizure medication are not to be dismissed as major negatively impacting contributors to social impairment in PWE. Drugs such as phenobarbital, topiramate, levetiracetam and zonisamide have been proven to exert a negative effect on mood causing anxiety and depression (Yang et al. 2020), suicidal tendencies, cognitive impairment, lower seizure-threshold and psychosis, predominantly when associated with other risk factors for neuropsychiatric imbalance such as homelessness.

Homeless people are affected by trimorbidity: physical illness, psychiatric disorders and substance abuse (Moss et al. 2021). Afflicted by various comorbid chronic illnesses (epilepsy, psychiatric disorders, alcoholism or substance abuse, etc.), these people are avid users of health services, mainly of ED, in acute flares of preventable ACSC. Without detailed clinical records of their epileptic seizures, every ED admission is addressed as a first episode and is extensively managed causing iatrogenic harm to the people. In an outpatient setting, homeless people reveal worse treatment adherence, follow up and number of seizure episodes, largely inadvertently caused by health services and volunteer associations themselves, leading to more disease burden and more difficulty to assure appropriate housing. Homeless people are known to report poorer quality of life, revealing worse overall health and three times more chronic diseases than housed people (Lewer et al. 2019). This group of people develops illnesses decades earlier than their average age of onset in the general population (Hwang 2001). Among the numerous diseases reported, homeless people showed higher prevalence of asthma, cardiac conditions, anxiety and, as previously mentioned, epilepsy (Hwang 2001; Lewer et al. 2019). Other dental and dermatological conditions were reported with a higher incidence in the homeless population, due to precarious sanitary living conditions and lack of appropriate footwear (Hwang 2001). Because of that insalubrious living conditions, lack of vaccination and everyday exposure, the homeless population is also especially vulnerable to Human Immunodeficiency Virus (HIV), neurocysticercosis, malaria, tuberculosis, among others infections, that might affect the brain and cause seizures (Scheffer et al. 2017).

Children of homeless families report an increased risk of developmental and mental health problems as well as epilepsy which go undetected for poor access to medical evaluation (Wood 1989). The severity of each reported illness can be abnormally high when combined with all the negative SDoH inherent to homelessness: extreme poverty, deferral in healthcare access, nonadherence to medical treatment and cognitive impairment (Hwang 2001). Most studies regarding health problems among the homeless population focus on its trimorbidity: the association of physical illness, psychiatric disorder, and substance abuse (Moss et al. 2021). Hwang (2001) reports that alcoholism is 6–7 times more prevalent in this population, with 60% rate of lifetime prevalence among homeless men. Alcoholism is a known risk factor for epilepsy. Lennard et al. (2022) studied a group of people with repeat hospital admissions after a seizure episode and encountered 12% of drug or alcohol induced seizures.

Numerous tools can be elaborated in order to prevent these alarming numbers of accidental and avoidable deaths in young people. Seizure-focused education for PWE can help limit the number of accidents and possible risks in everyday activities such as cooking and driving. Rimmer et al. (2007) developed a brochure in this sense to distribute to PWE and their relatives and increase awareness to burn prevention. Hwang (2001) suggests that Canada’s universal health insurance system is protective and responsible for the country’s lower mortality rates among homeless people, as there is an incentive for free disease prevention and harm reduction.

There are confounding factors regarding the prevalence of seizures and epilepsy in the homeless. High incidence of substance and alcohol abuse and withdrawal in this population can be both a cause and a risk factor for seizures. Same can be said for TBI, as a fall can be a consequence and a trigger for a seizure episode with no discernible radiographic evidence to support the timeline of events. This distinction between primary and secondary epilepsy is not always linear and can be over or underestimating the weight of these risk factors for homeless people suffering with seizures or epilepsy. Future studies shall look at different types of epilepsy and homeless categories, as respectively described by ILAE and *FEANTSA*.

Disease burden embodies the full impact of an illness or disorder. It sums morbidity, mortality and financial costs of a given disease, calculating the personal and economic toll that a disease can take in an individual’s life. The disease burden of epilepsy and homelessness combined is widely studied as investigators tend to notice the impact of this group as major consumers of health resources around the world: inpatient admissions, outpatient follow-up, treatment and mortality.

Homeless adults are frequent consumers of healthcare services, especially ED, with five times higher hospital admission rates and longer periods of hospitalization than general population (Hwang 2001). It is estimated that in a given group of 1000 homeless people, when in comparison with the same number of housed people, the homeless group would report 225 more ED admissions per year (Moss et al. 2021). Homeless people are often hospitalized for ACSC, a group of illnesses for which, through appropriate management in primary care outpatient settings (such as vaccinations and lifestyle interventions), it is possible to avoid acute exacerbations and prevent the need for hospital admission (O’Farrell et al. 2016).

Epilepsy also accounts for a big portion of health care costs and overall disease burden both for individuals and health services (WHO 2022). Homeless people living with epilepsy presented worse institutional quality measures such as hospital admissions, occupied hospital beds and unplanned readmissions, leading to worst outcomes for health centers globally (Ramamurthy et al. 2021). These people are being inappropriately managed in ED for ACSC, causing sub-optimal disease control, higher risk of future exacerbations and more economic burden for hospitals, as this is not a cost-effective strategy (Doran et al. 2021a, b). The risk of readmission in homeless people could be lessened by articulating and preparing a patient’s discharge with shelter providers including safe transportation (Greysen et al. 2012). Greysen et al. (2012) stated that 56% of homeless people were not inquired about their housing status while hospitalized and 42% of those who postponed seeking medical care did so out of fear of inability to find shelter after discharge. In fact, 11% of homeless people admitted to staying in the streets on the first night after hospital discharge, especially if they were discharged after dark. Signaling these repeat attendees and coding them for Homelessness and Epilepsy (QD71.0 and 8A60, respectively, in ICD-11), as well as known comorbidities such as substances abuse and psychiatric disorders, can help medical professionals identifying risk factors for worst outcomes (O’Toole et al*.* 2007; Rosendale et al. 2019; Doran et al. 2021a, b) and avoid discrepancies between future studies. To prevent and score the risk of hospital readmission in this group of people, healthcare services need to pay attention to every dimension of this illness: medical, social and personal. Public financing medical costs secondary to epilepsy treatment lifts the financial burden from those affected and is cost-effective, improving psychosocial stress and quality of life for PWE (WHO 2022).

Providing appropriate healthcare to homeless people is a difficult mission aggravated by seizures and epilepsy diagnoses. Without a way of being contacted (via telephone or fixed address) or to travel by car or paid public transportation an estimate 80% of homeless people do not attend their scheduled appointments in outpatient settings (Doran et al. 2021a, b). There are organizational barriers imposed by medical centers, shelters and volunteer associations that are contributing to this fact: inflexible schedules, facilities dispersed across large cities, health services jurisdictions and more; all of which are harming people whose living conditions are too precarious to be able to regularly attend specialized care services (Doran et al. 2021a, b; Moss et al. 2021). According to WHO (2022) up to 70% of PWE could live seizure-free if properly diagnosed and treated.

The development of epilepsy-awareness campaigns in the general population can be a simple yet effective measure to reduce the stigma associated with the disease and prevent social impairment, eventually leading to better outcomes in all SDoH. The stigma and discrimination that surround PWE are often more difficult to overcome than the seizures themselves (WHO 2022). Creating laws considering the Universal Declaration of Human Rights and holding governments accountable for its violations can have a big impact in the independence and overall quality of life of PWE. Designing scores for risk of homelessness based on known upstream factors (SDoH) and applying them in outpatient settings can protect people from housing instability and social disintegration (Rosendale et al. 2019).

It is fundamental that both hospitals and homeless shelters register people in open access Electronic Medical Records (EMR) to keep track of people’s clinical information, ED admissions and diagnoses and current housing situation to prevent duplication of medical exams and misinterpretation of clinical settings (such as recurring seizures) and consequently save resources (Booth 2006). To avoid iatrogenic harm, medical and volunteer teams providing services to homeless people should incorporate neurologists, to intervene at an early stage and properly treat seizures and epilepsy while potentially avoiding hospital admission (Rosendale et al. 2019). The ability to get medical prescriptions timely filled, store medications in safety and take them as prescribed is a big challenge to homeless people that should be taken in consideration while planning their follow-up (Subedi et al 2022). Medical experts, social workers, and volunteer teams should never dismiss the role of adequate nutrition, rest, social support and medical vigilance to the positive outcomes and increase of quality of life in homeless people suffering with epilepsy. Epilepsy, and seizures, among the homeless seems to be a strong argument to make an invitation to the most interested and motivated neurologists. Please, join psychiatrists working with these super difficult patients (Gama Marques 2021) on the streets of our cities, occupying the interstitial space between the traditional healthcare institutions. Join us on the plight of marontology (Gama Marques et al. 2023).

## Data Availability

Not available.
